# Proliferating cell nuclear antigen is required for loading of the SMCX/KMD5C histone demethylase onto chromatin

**DOI:** 10.1186/1756-8935-4-18

**Published:** 2011-10-13

**Authors:** Zhihui Liang, Marc Diamond, Johanna A Smith, Matthias Schnell, René Daniel

**Affiliations:** 1 Division of Infectious Diseases - Center for Human Virology, Department of Medicine, Thomas Jefferson University, Philadelphia, PA 19107, USA; 2 Department of Microbiology, Thomas Jefferson University, Philadelphia, PA 19107, USA; 3 Center for Stem Cell Biology and Regenerative Medicine, Thomas Jefferson University, Philadelphia, PA 19107, USA; 4 Kimmel Cancer Center, Immunology Program, Thomas Jefferson University, PA 19107, USA; 5 Infectious Diseases/Internal Medicine, Medical School of Yale University, WWW 419, 200S Frontage Rd, New Haven, CT 0651, USA; 6 Tufts Medical Center, 800 Washington St., number 401, Boston, MA 02111, USA

## Abstract

**Background:**

Histone methylation is regulated by a large number of histone methyltransferases and demethylases. The recently discovered SMCX/KMD5C demethylase has been shown to remove methyl residues from lysine 4 of histone H3 (H3K4), and constitutes an important component of the regulatory element-1-silencing transcription factor (REST) protein complex. However, little is known about the cellular mechanisms that control SMCX activity and intracellular trafficking.

**Results:**

In this study, we found that small interfering RNA-mediated knockdown of proliferating cell nuclear antigen (PCNA) resulted in the reduction of the chromatin-bound SMCX fraction. We identified a PCNA-interaction protein motif (PIP box) in the SMCX protein. Using site-directed mutagenesis, we found that the amino acids of the SMCX PIP box are involved in the association of SMCX with PCNA and its interaction with chromatin.

**Conclusions:**

Our data indicate that the intracellular trafficking of SMCX is controlled by its association with PCNA.

## Background

Cellular DNA in eukaryotes is organized in chromatin, which contains a multitude of proteins. Histone proteins comprise a major structural component of chromatin. Histones carry diverse post-translational modifications, particularly on the lysines of their N-terminus. These modifications include methylation, acetylation, ubiquitylation and phosphorylation [1-4]. Histone modifications are involved in crucial cellular processes, including transcription, DNA replication, and the DNA-damage response [1,3,5]. As might be expected, chromatin and histones undergo major reorganization during DNA replication. After replication, the appropriate chromatin structure has to be restored in order to maintain the passage of information from mother to daughter cells.

One of the major histone modifications is methylation of the lysine 4 residue of histone H3 (H3K4). Trimethylation of H3K4 is a hallmark of active transcription, and conversely, a loss of this modification represses transcription. Histone methylation is a very stable modification compared with, for example, acetylation. It had been long thought that this modification was irreversible [6]. This was supported by the fact that histone methyltransferases were identified, but their counterparts, histone demethylases, were not yet known. This situation changed with the first discovery of a histone demethylase, termed lysine-specific demethylase (LSD)1 [7]. LSD1 prefers double-methylated H3K4 as its substrate [7]. Following this discovery, many other histone demethylases were discovered, with various preferences for specific methylated histone residues. One of the newly discovered demethylases is SMCX (Smcy homolog, X-linked (mouse); also termed lysine-specific demethylase 5C (KDM5C) and jumonji, AT rich interactive domain 1C (JARID1C)) [8].

SMCX is a ubiquitously expressed protein, which has been linked to mental retardation [9-14]. In addition, a novel mutation in the *SMCX *gene was found in a patient with autism spectrum disorder (ASD) [15]. SMCX contains a JmjC domain, and belongs to a family of demethylases, which includes the Y-linked homolog SMCY, retinoblastoma binding protein (RBP)2, and; lysine-specific demethylase 5B (KDM5B; also termed PLU-1) [8,16]. In addition to JmjC, SMCX also contains the N-terminal plant homeodomain (PHD) finger, which binds to the trimethylated H3K4 (H3K4me3). SMCX reverses H3K4me3 to di- and mono-, but not unmethylated products [8]. SMCX was reported to function as a transcriptional repressor, and its loss impairs neuronal gene regulation mediated by regulatory element-1-silencing transcription factor (REST) [17,18]. However, little is known about how the SMCX-mediated demethylase activity is regulated during the cell cycle and other crucial cellular processes.

In this study, we found that SMCX-mediated H3K4 demethylation is regulated by proliferating cell nuclear antigen (PCNA). We show that association of SMCX with chromatin and H3K4me3 demethylation depends on PCNA. Further, we identified a aconserved sequence motif, [NQ]xx(L/I/M/V)xx (F/Y/W), called the PCNA-interaction protein motif (PIP box) in the SMCX protein. Finally, site-directed mutagenesis demonstrates that the PIP box is important for SMCX association with PCNA.

## Methods

### Cells, plasmids and PIP BOX mutants

We used 293T/17 cells (referred to as '293T' from this point onwards) (catalog number CRL-11268; American Type Culture Collection (ATCC) Manassas, VA, USA), which were maintained in DMEM medium supplemented with 10% FBS and antibiotics (Penn/Strep at 1:100).

A pCMV6-XL6 vector with SMCX cDNA was used (Origene Technologies, Rockville, MD, USA and expressed from the cytomegalovirus (CMV) promoter (number SC128277;). The PIP box in SMCX and G9a were identified using a ScanProsite search for conserved motifs in SMCX and G9a sequences. PIP box mutants were obtained by site-directed mutagenesis of the SMCX-encoding plasmid of residues F, L and Q, respectively, into A, and confirmed using sequencing and other standard methods.

### Transfections

Regularly maintained 293T cells were plated into cell-culture dishes with DMEM and 10% inactivated FBS, 1 day before transfection. The following day, the plates were transfected using Lipofectamine™ 2000 (number 11668027; Invitrogen Corp., Carlsbad, CA, USA), when transfected with small interfering (si)RNA and plasmid DNA, or Lipofectamine™ RNAiMAX (number 13778075; Invitrogen Corp.) when transfected with siRNA (targeting PCNA) alone.

### Small interfering RNAs

Pools of PCNA siRNA, G9a siRNA and the non-targeting control siRNA sequences were synthesized (numbers M-003289-02-0010, M-006937-01-0005 and P-001206-13-05 respectively; Dharmacon Research Inc., Lafayette, CO, USA). To deplete PCNA, 293T cells were transfected twice (Lipofectamine™ 2000) with the indicated siRNAs at 24-hour intervals. Cells were subsequently harvested at 48 hours after the first transfection, and lysed for immunoblotting experiments.

### Antibodies and beads

**Figure 1:**
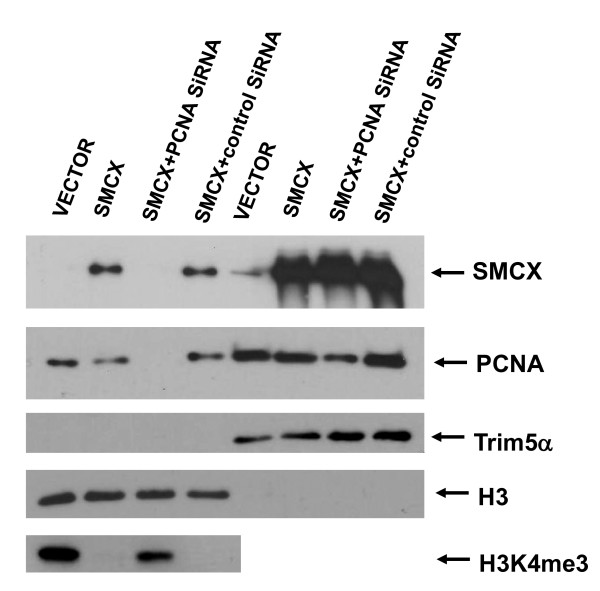
**Effect of proliferating cell nuclear antigen (PCNA) knockdown on the intracellular distribution of exogenous SMCX (Smcy homolog, X-linked (mouse)) protein**. 293T cells were transfected with the SMCX-encoding plasmid or the control empty vector, and PCNA or control small interfering (si)RNA. Two days after transfection, cells were harvested, then the chromatin fraction was separated from the rest of the lysate and the fractions analyzed by western blotting. The four samples on the left are of SMCX and PCNA levels in chromatin, and those on the right are SMCX levels in the whole-cell lysate without chromatin fraction. Histone H3 (chromatin protein) and TRim5α (cytoplasmic protein), respectively, were used as loading controls. The label 'Vector' indicates cells transfected with the empty plasmid vector.

**Figure 2:**
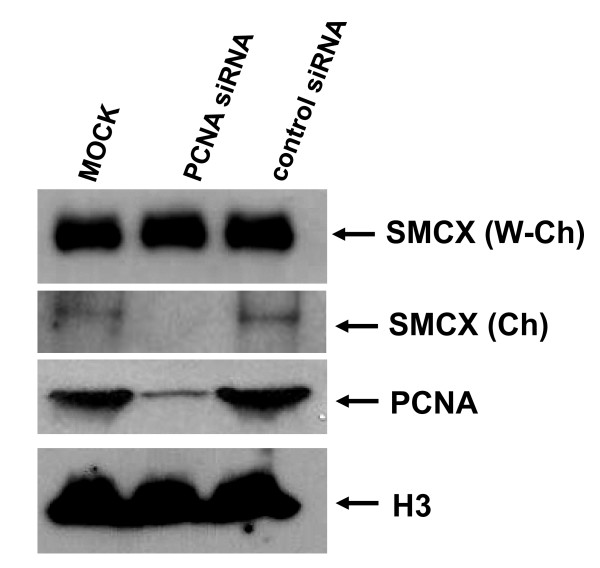
**Effect of proliferating cell nuclear antigen (PCNA) knockdown on intracellular distribution of endogenous SMCX (Smcy homolog, X-linked (mouse)) protein**. 293T cells were transfected with anti-PCNA or control small interfering (si)RNA. Two days after transfection, cells were harvested, then the chromatin fraction was separated from the rest of the lysate and fractions analyzed by western blotting. Ch = chromatin fraction, W-Ch = cell lysate without chromatin.

**Figure 3:**
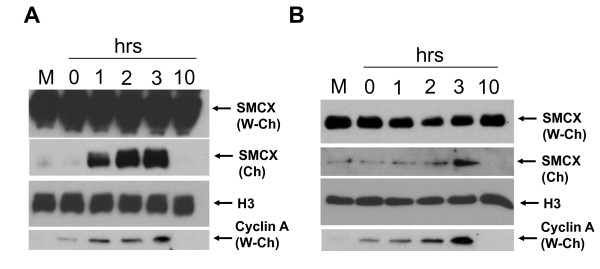
**Cell-cycle regulation of SMCX (Smcy homolog, X-linked (mouse))**. **(A) **SMCX levels in cells treated with and stained for (5-bromo-2'-deoxyuridine, BrdU). Cells were exposed to BrdU for 3 hours then double-stained with anti-BrdU and SMCX antibodies. Representative cells (high or low BrdU staining) are shown. Top two rows show cells exhibiting the granular pattern. **(B) **SMCX DNA was transfected into cells, and 2 days after transfection cells were synchronized with aphidicolin. One day later, cells were released from the aphidicolin block, and SMCX levels were monitored at indicated intervals using western blotting. **(C) **This experiment was designed as in (B), except that transfection was not performed and endogenous SMCX levels were monitored. Cyclin A = cyclin A levels, a cell-cycle synchronization control; cyclin A levels increase in late G1 and stay high through the S phase. H3 = histone H3, a loading control for the chromatin fraction. Other terminology as in Figure 5.

**Figure 4:**
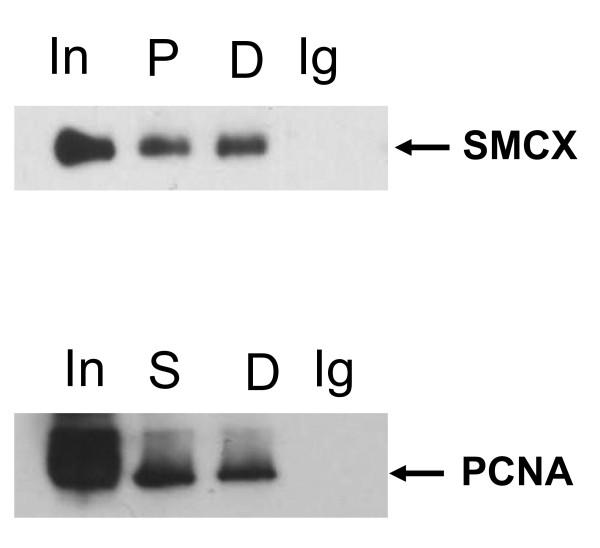
**Association of SMCX (Smcy homolog, X-linked (mouse)) and proliferating cell nuclear antigen (PCNA)**. 293T cells were transfected with the SMCX-encoding plasmid, and chromatin lysates prepared 2 days after transfection. Lysates were then immunoprecipitated with anti-SMCX, anti-PCNA, or control normal rabbit or mouse IgG, respectively. Some lysates were pretreated with DNAse, as described in Methods. SMCX and PCNA were detected by western blotting. D = DNAse-pretreated samples, immunnoprecipitated with the corresponding antibody, Ig = control normal IgG (mouse for the anti-PCNA antibody (top), and rabbit for the anti-SMCX antibody (bottom)), In = input lysate, P = samples immunoprecipitated with the anti-PCNA antibody, S = samples immunoprecpitated with the anti-SMCX antibody.

**Figure 5:**
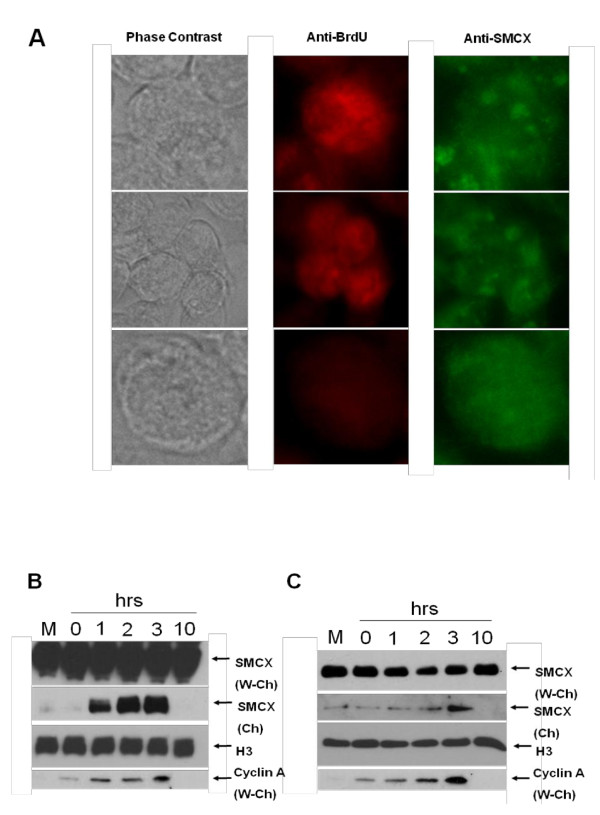
**Association with proliferating cell nuclear antigen (PCNA) and Intracellular distribution of SMCX (Smcy homolog, X-linked (mouse)) PCNA-interaction protein motif (PIP box) mutants**. **(A) **Intracellular distribution of PIP mutants. 293T cells were transfected with plasmid encoding SMCX and mutated SMCX Two days after transfection, cells were harvested, then the chromatin fraction was separated from the rest of the lysate and both fractions analyzed by western blotting. SMCX mutants in chromatin: SMCX = normal SMCX, F1209A = F to A mutant (see text), L1204A = L to A mutant, Q1201A = Q to A mutant. H3 and GAPDH served as loading controls. Ch = chromatin fraction, W-Ch = cell lysate without chromatin (bottom), **(B) **Association of PIP mutants with PCNA 293T cells were transfected as in (A). Two days after transfection, cells were harvested, whole-cell lysates prepared and immunoprecipitated with PCNA. SMCX and PIP mutants in immunoprecipitates were detected using western blotting. Terminology as in (A).

The antibodies used were: anti-histone H3 (number ab1791-100), anti-SMCX (number ab72152 for Figures 1, 2 and 3, and ab34718 for Figures 4 and 5), a and anti-H3K4me3 (number ab8580) (all Abcam, Cambridge, Cambridgeshire, UK); anti-PCNA (number sc-56), Trim5 (number sc-48319) and anti-GAPDH (number sc-2037) (all Santa Cruz Biotechnology Inc., Santa Cruz, CA, USA); Anti-BrdU conjugated to Alexa Fluor 594 (number A21304) and secondary donkey anti-rabbit conjugated to Alexa Fluor 488 (number A21206) (both Invitrogen Corp).

### Preparation of chromatin fractions and western blotting

The cells were washed with PBS once, then pelleted by centrifugation. Pellets were resuspended in lysis buffer with 1% Triton X-100 and fresh protease inhibitors, and incubated on ice for 5 minutes. Lysates were then separated by centrifugation at 13,000 rpm for 5 minutes. The resulting supernatant contained cytoplasmic and nuclear proteins without the chromatin fraction. The chromatin-containing pellet was resuspended in the sample buffer containing SDS, and denatured by heating at 90°C for 10 minutes; it was then incubated on ice for 5 minutes. Before loading onto SDS-PAGE gels, samples were treated with sonication to break up DNA, and separated by centrifugation. After electrophoresis, samples were transferred to a membrane (Immobilon P; number IPVH00010; Millipore, Billerica, MA, USA), and incubated with primary and secondary antibodies in 5% milk. The Santa Cruz antibodies were diluted 1:200, and all other antibodies were diluted 1:1000. All secondary antibodies were diluted 1:5000. Bands were detected using chemiluminescence.

### Immunoprecipitation

The cells were harvested and chromatin lysates prepared as described previously [19]. The immunoprecipitation was carried out at 4°C overnight by incubating the lysates with the primary antibody of interest or a normal IgG (Santa Cruz, rabbit IgG sc2027, mouse IgG sc-2025). The after day, protein-antibody complexes were isolated by binding to protein G plus/protein A agarose (number IP05; Calbiochem-Novabiochem International, Inc., La Jolla, CA, USA). for 1 hour at 4°C. Immunoprecipitates were collected and washed 3 times with the lysis buffer, resuspended in the SDS sample buffer and heated for 5 minutes at 90°C. The protein G plus/protein A agarose was removed by centrifugation. The samples were then loaded onto SDS-PAGE and processed as described above for western blotting. The DNAse pre-treatment was performed as described previously [19].

### Immunofluorescence

293T cells were plated onto four-well slides (Lab Tek Chamber;Thermo Scientific, Rochestyer NY)] at a density of 4 × 10^5 ^per well. The next day cells were exposed to BrdU for 3 hours at a concentration of 10 μmol/L. Cells were washed once with PBS and then fixed with cold methanol for 20 minutes. DNA was then denatured by two consecutive treatments with 4 mol/L HCL for 15 minutes each. Cells were then washed with PBS and incubated with antibodies diluted 1:50 in PBS with 3% BSA (anti-BrdU conjugated to Alexa Fluor 594 and anti-SMCX, or a control with no antibody) overnight at 4°C. The next day, cells were washed with PBS containing Tween 20, and then incubated with a secondary donkey anti-rabbit antibody targeting SMCX (controls consisted of a well with no antibody, or a well with secondary antibody alone; data not shown) in PBS with 3% BSA for 1 hour at room temperature. Cells were then washed with PBS with 3% BSA, then incubated with DAPI for 5 minutes, then mounted with antifade reagent (number P36930; ProLong^® ^Gold; Invitrogen Corp.) and cured overnight before pictures were taken (Eclipse TE-2000S; Nikon, Tokyo, Japan).

### Synchronization experiments

293T cells were plated onto 60 mm dishes and tranfected with the SMCX plasmid as described above. Two days after transfection, aphidicolin was added to a final concentration of 5 mg/ml. One day later, the aphidicolin-containing medium was removed and cells washed. Standard medium was added, then cells were harvested at the indicated intervals and analyzed by western blotting. The experiment described in Figure 3C was performed in a similar manner, except that cells were not transfected with the SMCX plasmid.

## Results

### Association of overexpressed SMCX in chromatin depends on proliferating cell nuclear antigen

Because endogenous SMCX expression is reported to be low in most types of somatic cells, except neurons [8], we initially used an overexpression approach and transiently overexpressed SMCX under control of the CMV promoter. Overexpressed SMCX was found to be present in both whole-cell lysates and the chromatin fraction (Figure 1), but the levels in chromatin, although still significant, were lower than those in the whole-cell lysates.

We then treated cells with siRNA targeting PCNA. PCNA knockdown strikingly reduced the amount of SMCX in chromatin (Figure 1). Control siRNA did not affect SMCX levels in the chromatin fraction, and PCNA siRNA did not significantly affect the overall SMCX levels in the whole-cell lysate (results were quantified by densitometry, data not shown). H3K4me3 levels inversely correlated with SMCX levels in chromatin. Our results thus show that PCNA is required for the entry of exogenous SMCX into chromatin.

### Association of endogenous SMCX with chromatin also depends on PCNA

293T cells do express SMCX, although at very low levels. Large amounts of protein lysate are required to detect endogenous SMCX by western blotting. Thus, to investigate whether our conclusions can be extended to endogenous SMCX, we transfected 293T cells with PCNA siRNA, and examined the levels of endogenous SMCX in chromatin. DNA knockdown also reduced levels of endogenous SMCX (Figure 2). This result also supports our initial conclusion that PCNA is required for SMCX association with chromatin.

### SMCX associates with proliferating cell nuclear antigen

PCNA carries binding sites for a large number of transcription factors and other chromatin-associated proteins [20]. We hypothesized that SMCX may associate with PCNA, similar to other proteins that affect chromatin structure. To test this hypothesis, we performed co-immunoprecipitation experiments and analyzed SMCX levels in PCNA immunoprecipitates of the chromatin fraction and *vice versa*. SMCX co-immunoprecipitates with PCNA (Figure 4), and thus we conclude that SMCX associates with PCNA.

We also tested the hypothesis that the PCNA-SMCX interaction is mediated by DNA. Thus, samples were pretreated by DNAse, as described previously [19]. The treatment did not affect the levels of co-immunoprecipitated SMCX or PCNA., indicating that SMCX-PCNA co-immunoprecipitation reflects protein-protein interaction.

### SMCX contains a PIP box

The PIP box,[NQ]xx(L/I/M/V)xx (F/Y/W), is found in some PCNA-interacting proteins, such as the p21 (WAF1/CIP1) protein [20,21], and it interacts with the interdomain connector loop (IDCL) of PCNA. Because PCNA is a homotrimer, it is possible that complexes containing up to three PCNA-interacting proteins can be assembled on a single PCNA ring. For example, each of the subunits in a human PCNA trimer can simultaneously bind to a different flap endonuclease (FEN)-1 molecule [22,23].

**Figure 6:**
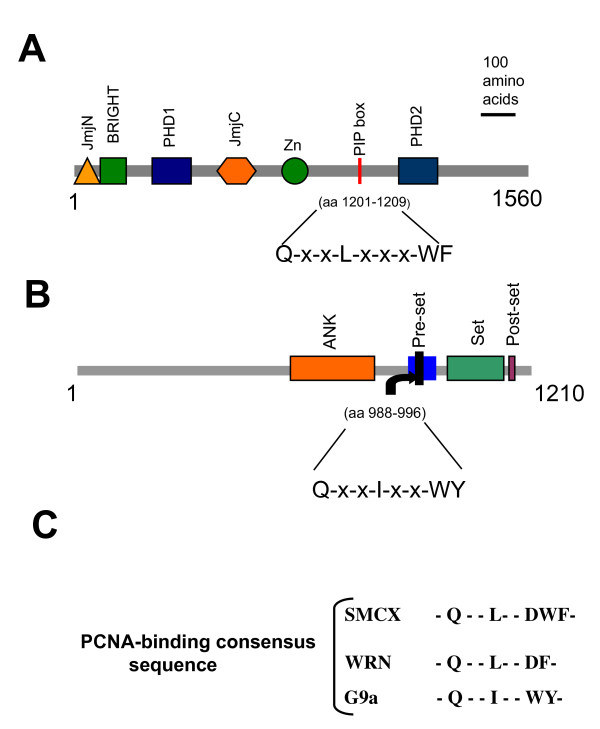
**Profliferating cell nuclear antigen-interaction protein motif (PIP box) boxes in SMCX (Smcy homolog, X-linked (mouse)) and G9a. PIP boxes were identified as described in Methods. (A) SMCX; (B) G9a; (C) comparison of SMCX, G9a and WRN (Werner syndrome protein) PIP boxes**. SMCX domains: BRIGHT = DNA binding domain, JmjC = JmjC domain, JmjN = JmjN domain, PHD1 = PHD-finger 1, PHD2 = PHD-finger 2, Zn = zinc finger. G9a domains: ANK = Ankyrin repeats domain, Pre-set = Pre-set motif, Post-set = Post-set domain, Set = Set domain.

Another novel PCNA-binding motif, K-A-(A/L/I)-(A/L/Q)-x-x-(L/V), is termed the KA box. It is distinct from the 'classic' PIP box and is also present in several PCNA-interacting proteins [20]. To determine if SMCX contains either of these PCNA-interacting motifs, we performed an analysis of the SMCX protein sequence using the ScanProsite program. We identified a PIP box in the SMCX protein (Figure 6A): - - Q- - L- - -WF- -. The full amino acid sequence is: - -QCDLCQDWF- -.

### PIP box in proteins of the regulatory element-1-silencing transcription factor complex

We then tested a hypothesis that SMCX association with PCNA is indirect, and is mediated by another protein, rather than the SMCX PIP box. Because SMCX is a component of a protein complex that contains REST, the histone deacetylases HDAC1 and HDAC2, and the histone methyltransferase G9a, we analyzed these proteins for the presence of a PIP box motif, and found one in the G9a protein (Figure 6B). The motif is of the type found in SMCX. This finding prompted tanother question: does PCNA bring SMCX into chromatin directly, or is SMCX brought into chromatin by interacting with G9a, which in turn interacts directly with PCNA?

To address this issue, we knocked down G9a in 293T cells using siRNA. We found that G9a knockdown did not affect chromatin levels of SMCX (see Additional file 1, Figure S1), supporting the hypothesis that the entry of SMCX into chromatin is due to direct interaction with PCNA.

### SMCX PIP box mutants exhibit reduced association with chromatin

To test the hypothesis that the putative SMCX PIP box mediates the interaction with PCNA and loading SMCX onto chromatin, we mutated three of the conserved residues within the PIP box (F, L and Q; Figure 6), and investigated whether these mutant proteins associate with PCNA and chromatin. We found that mutations of F to A, L to A and Q to A all led to reduced co-immunoprecipitation of SMCX and PCNA (Figure 5B), Similarly, all three mutants were reduced in chromatin compared with normal SMCX (Figure 5A). We conclude that the amino acids of the SMCX PIP box control the interaction of SMCX with PCNA and the loading of SMCX onto chromatin.

### SMCX association with proliferating cell nuclear antigen is regulated by the cell cycle

PCNA is mainly expressed in S phase. To determine if SMCX levels similarly fluctuate inside cells during the cell cycle, we performed immunofluorescence experiments, and found that SMCX was expressed both in cells with strong staining with anti-BrdU antibody (S phase) and in cells showing very little BrdU staining (Figure 3A). However, SMCX in S phase cells had a different, granular pattern of distribution compared with other cells. This result suggests a possible SMCX redistribution in S phase.

Cells were then synchronized with aphidicolin at the G1/S interface, and released by removing the drug. Both exogenous and endogenous SMCX had very high upregulation in the chromatin fraction after entry into S phase (Figure 3B, C). We thus conclude that SMCX is present in chromatin mainly in S phase, which correlates with the expression pattern of PCNA.

## Discussion

SMCX/JARID1C is a recently discovered H3K4 demethylase, which belongs to a family of JARID1 proteins that also contains the demethylases SMCY (JARID1D), RBP2 (JARID1A) and PLU1 (JARID1C). These two latter demethylases share SMCX preferences for H3K4me3 and H3K4me2 [8]. However, the *SMCX *gene was identified before the discovery of its demethylase activity and its mutations were associated with X-linked mental retardation (XMLR), which affects 1 in 500 males. This gene is also one of the few that escape from inactivation of the mammalian X chromosome [9]. A novel mutation was found recently in the *SMCX *gene of a patient with ASD, and it may be responsible for producing the autism phenotype in this patient [15]. It was thus thought that the SMCX role is restricted to neurons and, it was consistently shown that SMCX expression during zebrafish development is highest in the brain, and that SMCX is required for neuron survival and dendritic development [8].

Nevertheless, other results argue for an additional role of SMCX in non-neuronal cells. SMCX was found to be ubiquitously expressed during early mammalian development [8], and it is also involved in E2-mediated repression of papillomavirus oncogenes. In addition, SMCX levels were found to be increased in prostate and seminoma cancers and to be absent in renal carcinomas [24,25]. Finally, SMCX was found in a protein complex with REST, which represses transcription of neuronal genes in non-neuronal tissues, and was recently found to play a tumor suppressor role in breast cancer by suppression of the oncogenic TAC1 (tachykinin, precursor 1) protein [7,26]. These results support a role for SMCX in non-neuronal, dividing cells.

SMCX is one of the few known H3K4 demethylases, and H3K4 methylation is a crucial epigenetic modification that regulates both transcription and the DNA-damage response [27,28]. In addition, SMCX has been shown to play a role in disease development (see above). It is thus crucial to understand the cellular regulation of SMCX function. Unfortunately, very little is known about how this gene and protein are regulated. Some information can be inferred from yeast, which contains the SMCX homolog, Jhd2 [29]. Levels of Jhd2 protein in yeast are modulated through polyubiquitylation by the E3 ubiquitin ligase Not4 and its turnover is mediated by the proteasome [30]. Polyubiquitin-mediated degradation of Jhd2 controls *in vivo *H3K4 trimethylation and gene-expression levels. Human NOT4 can also polyubiquitylate JARID1C/SMCX, a human homolog of Jhd2, suggesting that this is probably a conserved mechanism [30].

In this study, we showed that SMCX entry into chromatin depends on PCNA, which is found in complex with SMCX. SMCX contains a PIP box, whose amino acids are important for the association of SMCX with PCNA and chromatin. PCNA is expressed by dividing cells, predominantly in the S phase of the cell cycle, when it associates with replication forks [31]. Our results support a role for SMCX in dividing cells, in addition to neurons, possibly in the establishment and maintenance of H3K4 methylation patterns during and after the S phase of the cell cycle.

## Conclusions

In summary, our data indicate that the intracellular trafficking of SMCX is controlled by its association with PCNA.

## List of abbreviations

DMEM: Dulbecco's modified Eagle's medium; DAPI: 4',6-diamidino-2-phenylindole; FBS: fetal bovine serum; GADPH: glyceraldehyde 3-phosphate dehydrogenase; PBS: phosphate-buffered saline; SDS-PAGE: sodium dodecyl sulphate polyacrylamide gel electrophoresis

## Competing interests

The authors declare that they have no competing interests.

## Authors' contributions

ZL made the crucial discovery, that SMCX associates with PCNA and PCNA is required for SMCX association with chromatin performed the experiments indicating that SMCX import into chromatin is controlled by PCNA, and identified the SMCX PIP box. MD created the PIP box mutants, JAS performed the immunofluorescence experiments, and MS was involved in writing of the manuscript and experimental design. RD wrote the manuscript and also performed western blot experiments. All authors read and approved the final manuscript.

## Supplementary Material

Additional file 1 **Figure S1. Effects of G9a knockdown on chromatin levels of SMCX (Smcy homolog, X-linked (mouse))**. 293T cells were transfected with the SMCX-encoding plasmid and anti-G9a, anti-proliferating cell nuclear antigen, or control small interfering RNA. Two days after transfection, cells were harvested. The chromatin fraction was separated from the rest of the lysate, and analyzed by western blotting. Click here for file
