# Supplementary figures and images for: Proliferating cell nuclear antigen is required for loading of the SMCX/KMD5C histone demethylase onto chromatin
Source: Epigenetics Chromatin. 2011 Oct 13;4:18. doi: 10.1186/1756-8935-4-18 (PMC3212929; doi:10.1186/1756-8935-4-18)

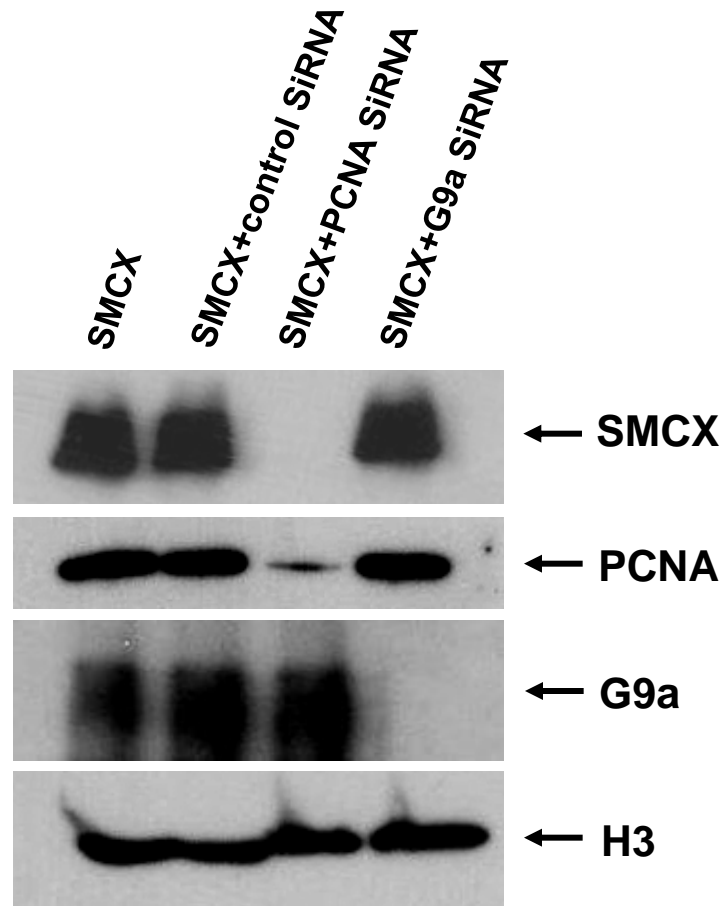

Figure S1

Supplement: Additional file 1 — Figure S1. Effects of G9a knockdown on chromatin levels of SMCX (Smcy homolog, X-linked (mouse)). 293T cells were transfected with the SMCX-encoding plasmid and anti-G9a, anti-proliferating cell nuclear antigen, or control small interfering RNA. Two days after transfection, cells were harvested. The chromatin fraction was separated from the rest of the lysate, and analyzed by western blotting. [file 1756-8935-4-18-S1.PDF]
